# Immune Parameters for Diagnosis and Treatment Monitoring in Invasive Mold Infection

**DOI:** 10.3390/jof5040116

**Published:** 2019-12-16

**Authors:** Jeffrey D. Jenks, Stephen A Rawlings, Carol Garcia-Vidal, Philipp Koehler, Toine Mercier, Juergen Prattes, Cornelia Lass-Flörl, M Teresa Martin-Gomez, Dieter Buchheidt, Livio Pagano, Jean-Pierre Gangneux, Frank L. van de Veerdonk, Mihai G. Netea, Agostinho Carvalho, Martin Hoenigl

**Affiliations:** 1 Department of Medicine, University of California San Diego, San Diego, CA 92103, USA; 2 Clinical and Translational Fungal—Working Group, University of California San Diego, La Jolla, CA 92093, USA; strawlings@ucsd.edu; 3 Division of Infectious Diseases and Global Public Health, Department of Medicine, University of California San Diego, San Diego, CA 92093, USA; 4 Infectious Diseases Department, Hospital Clinic-IDIBAPS, 08036 Barcelona, Spain; carolgv75@hotmail.com; 5 University of Cologne, Faculty of Medicine, Department I of Internal Medicine, Center for Integrated Oncology Aachen Bonn Cologne Duesseldorf (CIO ABCD), Excellence Center for Medical Mycology (ECMM), University Hospital of Cologne, 50931 Cologne, Germany; philipp.koehler@uk-koeln.de; 6 Cologne Excellence Cluster on Cellular Stress Responses in Aging-Associated Diseases (CECAD), University of Cologne, 50931 Cologne, Germany; 7 Department of Hematology, University Hospitals Leuven, 3000 Leuven, Belgium; toine.mercier@kuleuven.be; 8 Section of Infectious Diseases and Tropical Medicine and Division of Pulmonology, Medical University of Graz, 8036 Graz, Austria; Juergen.prattes@medunigraz.at; 9 Department of Microbiology and Hygiene, Medical University of Innsbruck, 6020 Innsbruck, Austria; cornelia.lass-floerl@i-med.ac.at; 10 Department of Microbiology, Vall d’Hebron University Hospital, 08035 Barcelona, Spain; mtmartin@vhebron.net; 11 Department of Hematology and Oncology, University Hospital Mannheim, Heidelberg University, 68167 Mannheim, Germany; dieter.buchheidt@umm.de; 12 Department of Hematology, Università Cattolica del Sacro Cuore, 00168 Rome, Italy; livio.Pagano@unicatt.it; 13 CHU de Rennes, Univ Rennes, Inserm, Irset (Institut de recherche en santé, environnement et travail)—UMR_S, 1085 Rennes, France; jean-pierre.gangneux@univ-rennes1.fr; 14 Department of Internal Medicine, Radboud University Medical Center, 6525 Nijmegen, The Netherlands; frank.vandeveerdonk@radboudumc.nl (F.L.v.d.V.); mihai.netea@radboudumc.nl (M.G.N.); 15 Life and Health Sciences Research Institute (ICVS), School of Medicine, University of Minho, 4710-057 Braga, Portugal; agostinhocarvalho@med.uminho.pt; 16 ICVS/3B’s—PT Government Associate Laboratory, University of Minho, 4710-057 Braga, Portugal

**Keywords:** *Aspergillus*, invasive aspergillosis, invasive mold infections, immune response, Mucormycosis, prognosis, mold-reactive T-cells, interleukin 6, interleukin 8, interleukin 10

## Abstract

Infections caused by invasive molds, including Aspergillus spp., can be difficult to diagnose and remain associated with high morbidity and mortality. Thus, early diagnosis and targeted systemic antifungal treatment remains the most important predictive factor for a successful outcome in immunocompromised individuals with invasive mold infections. Diagnosis remains difficult due to low sensitivities of diagnostic tests including culture and other mycological tests for mold pathogens, particularly in patients on mold-active antifungal prophylaxis. As a result, antifungal treatment is rarely targeted and reliable markers for treatment monitoring and outcome prediction are missing. Thus, there is a need for improved markers to diagnose invasive mold infections, monitor response to treatment, and assist in determining when antifungal therapy should be escalated, switched, or can be stopped. This review focuses on the role of immunologic markers and specifically cytokines in diagnosis and treatment monitoring of invasive mold infections.

## 1. Introduction

Invasive mold infections (IMI) continue to be associated with significant morbidity and high mortality rates globally, particularly in immunocompromised individuals [1,2,3,4]. Early diagnosis and appropriate treatment remain the most important predictors of survival [5], although early diagnosis remains difficult to establish, particularly at the initial stages of infection [6,7], and treatment is becoming increasingly complicated due to antifungal resistance [8,9].

Mycological diagnosis of IMI is challenging, with many molds not growing in blood cultures and cultures of bronchoalveolar lavage fluid (BALF) having a low sensitivity [10,11,12]. Consequently, fungal biomarkers such as galactomannan (GM), (1,3)-β-d-glucan (BDG), and extracellular glycoprotein, as well as molecular diagnostic tests (polymerase chain reaction (PCR)) applied to blood or bronchoalveolar lavage fluid (BALF), have emerged [7,11,13,14,15,16]. However, performance of these biomarkers and diagnostic tests has been shown to be less than optimal for invasive aspergillosis (IA) in patients receiving mold-active prophylaxis or treatment, which has been shown to reduce sensitivity of diagnostic tests [13,17,18,19,20,21,22]. In addition, sensitivity for molecular blood tests varies based on the degree of immunosuppression and neutrophil count, which triggers angioinvasive versus airway invasive growth in IA [23,24]. Specific biomarkers are also lacking for most other invasive mold infections, including mucormycosis, lomentosporiosis, and scedosporiosis [1]. In addition, cytokines are adjunctive tests to be used along with other markers to establish the etiology, as some of them also increase in non-fungal infections.

The performance of currently available biomarkers for the early diagnosis of IMI may be enhanced by combining multiple biomarkers and immunologic signals to maximize sensitivity and specificity [9]. Various molds have been shown to induce T-helper cell (Th)1 and Th17 subsets, resulting in elevated levels of several cytokines [25,26,27]. As cytokines are centrally involved in protective immunity against Aspergillus spp. and other molds [28,29], they have been a target for potential immunologic markers for the diagnosis of IMI [9].

Given that immunologic markers in blood have shown promise in the diagnosis of IMI, they may also serve as candidates for early assessment of treatment efficacy and antifungal treatment stratification. Previous studies have shown that serum GM kinetics may offer the clinician a substantial support in decision-making concerning early change, intensification, or even discontinuation of antifungal therapy, and may therefore facilitate therapeutic management of patients with IA [30,31]. In particular, persistently positive GM values may warrant further clinical/radiological evaluation to evaluate for breakthrough IMI and, if increasing, prompt early escalation of anti-mold therapy [31]. Importantly, serum GM has limited sensitivity, particularly in individuals without neutropenia or those on antifungal prophylaxis, and is only specific for Aspergillus spp. [30]. It is produced by very few other molds such as Fusarium spp. [32], reducing the applicability of GM kinetics for treatment monitoring in patients with non-Aspergillus IMI. Immunologic markers may therefore be particularly valuable for patients with invasive aspergillosis and negative serum GM levels and for patients with other IMIs where reliable blood biomarkers are lacking, although cytokine levels may vary among different patient groups (e.g., SOT patients or patients with chronic respiratory disease on chronic steroids) and further understanding of the performance of these immunologic markers across different patient groups is needed.

Here, we review the literature on immunologic marker for the diagnosis and treatment monitoring of IMI.

## 2. Anti-Mold Immune Response: The Impact of the Host and the Pathogen and Translational Implications

Studies have shown that cytokines are centrally involved in protective immunity against Aspergillus spp. and other molds [28,29]. While dormant, Aspergillus spores do not induce an inflammatory response. Once germination occurs in the host, the innate immune response is triggered [33,34] and ciliated epithelium, alveolar macrophages, and dendritic cells serve as the first line of defense against germlings [35], driving the production of cytokines and chemokines that direct the innate and adaptive immune responses [36]. In the early stages of IA, conidia are neutralized by local alveolar macrophages, and IL-8, a known neutrophil chemotactic factor, is produced by macrophages and epithelial cells to attract neutrophils to the site of infection [25,29]. In vitro studies have linked the increased release of IL-8 to the up-regulation of gene transcription by A. fumigatus [36]. These studies also found increased production of IL-6 by A549 pulmonary epithelial cells and primary epithelial cells [37], and this is thought to play an important role in T-cell recruitment. Other studies have shown that in vitro opsonization of *A. fumigatus* conidia with H-ficolin, L-ficolin [38], and M-ficolin play essential roles in pathogen recognition and complement activation through the lectin pathway and potentiate IL-8 secretion by A549 lung epithelial cells [39,40].

The immunologic response to invading conidia is not homogeneous, and the risk of IA is thought to be related to genetic factors and levels of expression or functional activity of cytokines and chemokines [41]. The risk of IA has been associated with deficiency in the (1,3)-β-d-glucan receptor dectin-1 [42,43,44], Toll-like receptor 4 (TLR4) polymorphisms [45,46], increased damage-associated molecular pattern (DAMP) signaling [47], decreased chemokine (C-X-C motif) ligand 10 (CXCL10) expression [48], DECTIN1 Y238X polymorphism [43], and polymorphisms for the pentraxin-3 gene (PTX3) [49,50,51,52].

Once exposed to Aspergillus conidia, the incubation period prior to invasion and clinical signs and symptoms of infection is not well defined but is likely highly variable based on the burden of inhaled conidia and underlying host characteristics, especially the degree of immunosuppression. One analysis of individuals with acute myeloid leukemia (AML) estimated the median incubation period of IA at 14.6 days (12.8–16.5 days), although this estimate was based on the time interval between the onset of severe neutropenia and diagnosis of IA [53]. Another study evaluated Aspergillus airborne spore counts and compared these levels to the diagnosis of IA in hospitalized patients and found that IA diagnosis was associated with high airborne spore counts in the preceding period from 28 to 42 days [54]. In addition, in this study, hospital admission for IA was associated with the presence of circulating respiratory viruses, particularly respiratory syncytial virus, adenovirus, and the H1N1 strain of Influenza A. The suspected incubation period in patients with severe influenza and IA in one study was significantly shorter given that the median time to IA diagnosis was 3 days [55]. Further attempts at evaluating incubation period of IA and other IMIs would be instructive.

Although many similarities exist between infections from Aspergillus spp. and other pathogenic filamentous fungi in terms of biology, risk factors, and clinical presentation, the host immune response varies. In vitro, *Rhizopus oryzae* has been shown to induce significantly higher levels of IL-6 and tumor necrosis factor alpha (TNFα) release from healthy human mononuclear cells, when compared to Aspergillus spp. [56]. In a model of invasive Mucorales infection, peripheral blood mononuclear cells (PBMCs), monocytes, and monocyte-derived dendritic cells (mDCs) from healthy donors were stimulated with resting and germinated stages of Mucorales and *Ascomycota*. Both inactivated germ tubes and resting spores of Mucorales species significantly stimulated mRNA synthesis and secretion of proinflammatory cytokines, including IL-6 and TNFα, and induced the upregulation of co-stimulatory molecules on mDCs and a T-helper cell response [57]. Another model of stimulated mononuclear cells examined TNFα and IL-6 secretion to cells exposed to different pathogenic fungi. *Lomentospora prolificans* induced significantly higher levels of TNFα and IL-6 secretion, compared to *A. fumigatus*-exposed cells, while *R. oryzae* caused release of higher levels of TNFα, compared to cells exposed to the Aspergillus spp., and IL-6 levels were very high as well, but when compared to the other fungi these levels were not statistically different [58].

Thus, fungal pathogens trigger a specific immune response that may be measurable before clinical signs and symptoms of disease develop, and may aid diagnosis and treatment monitoring in a field where available gold-standard diagnostics are imperfect.

## 3. Immunologic Markers for Diagnosis of Invasive Mold Infections

As early diagnosis of IA and IMI is associated with improved outcomes [5], more rapid diagnosis is crucial and identifying immunologic markers for early diagnosis may lead to decreased morbidity and mortality in IMI with improved outcomes.

Table 1 gives an overview of immunologic markers for diagnosis of IMI. In a prospective, nested, case-control study of adults with underlying hematological malignancy and suspected pulmonary infection, a bundle of cytokines was tested from BALF and serum to investigate whether any cytokines were significantly associated with IPA. Compared to matched controls, levels of IL-8 and IL-6 were significantly higher in serum and BALF among individuals with IPA [28]. In another nested, case-control study from Belgium involving 48 patients with probable or proven IA and 48 matched controls, of which each 30 had underlying hematological malignancies, a panel of biomarkers from serum and BALF was obtained, and IL-8, followed by IL-6 and IL-23 were best at discriminating IPA from no IPA in BALF, with IL-8 alveolar levels ≥ 904 pg/mL predicting IPA with high sensitivity (90%), specificity (73%), and negative predictive value (88%) [40]. In that study, serum levels of IL-6, IL-8, IL-17A, and IL-23 were also significantly increased among patients with IPA [40].

In addition, a recent single-center cohort study from Austria found that in a high-risk cohort of adult patients with underlying hematological malignancy and suspected IPA, of which the majority were receiving anti-mold prophylaxis at the time of BALF and serum sampling, IL-8 was the most reliable blood biomarker in those with IA and IMI, compared to controls, when a high cut-off was used, and exhibited close-to-perfect performance when combined with either the BALF Lateral Flow Device Test (LFD) or BALF Aspergillus PCR [9]. The cohort study design and the collection of same-day serum samples in this study allowed the investigators to try to determine the “real-life” diagnostic potential of these cytokines for diagnosing IPA and IMI, without having to take into account numerous covariates, and to evaluate combinations of biomarkers such as IL-8 with other diagnostic tests, such as BALF PCR and BALF LFD. Importantly, these analyses confirmed the potential clinical value of IL-8 in diagnosing IA in patients with hematologic malignancy, and to a lesser extent non-Aspergillus IMI, specifically when combined with the BALF LFD or BALF PCR or other more specific biomarkers of IA. Limitations of the studies include the small number of proven cases of IA and the fact that they included very few cases of other IMI. For example, in the Austrian study, of 106 cases of suspected pulmonary IA, there were only 11 probable and 32 possible cases of IA [9]. The low numbers of proven/probable IMI were hypothesized to reflect the use of highly effective anti-mold prophylaxis strategies in place at the center performing the study. Overall, IMIs are rare in patients receiving anti-mold prophylaxis (2%–3% prevalence) [59,60,61] and therefore multicenter studies are needed to confirm these findings in larger cohorts of patients with IA and other IMI.

## 4. Immunologic Markers for Treatment Monitoring in Invasive Mold Infections

Immunologic markers could be helpful to assist treatment stratification in predicting treatment outcomes. They may help predicting the likelihood of a favorable treatment response versus risk of disease progression or relapse, or even breakthrough infection [62]. They may also assist in the transition from intravenous to oral therapy and offer guidance in treatment cessation. 

Table 1 gives an overview of immunologic markers for treatment monitoring of IMI. In a model of neutropenic rats infected with IA and treated with amphotericin B, there was an association with decreased levels of GM in the lungs and serum and decreased levels of IL-6, macrophage Inflammatory protein 2 (MIP-2), and monocyte chemoattractant protein 1 (MCP-1) in the lungs, likely reflecting successful treatment and decreased fungal loads and inflammation [63]. In humans with IA and a variety of immunocompromising conditions, multiple cytokines, particularly IL-6 and IL-8, have shown promise for treatment monitoring and outcome prediction. In a multi-center study of 119 patients with IA, IL-6, IL-8, and IL-10 levels were generally elevated at baseline and trended down over time with treatment [64]. Persistently elevated levels of circulating IL-6 and CRP at week 1 were associated with treatment failure and death. Also, high levels of IL-8 at baseline were associated with poor clinical response and death at week 12 (sensitivity of 85% and specificity of 55% using a cutoff >135 pg/mL). In addition, persistently elevated levels of IL-6 and IL-8 during serial weekly measurements were associated with poor outcome. Conversely, sharp declines in IL-10 within the first 2 weeks of therapy were associated with lack of response to treatment [64].

Serum and BALF were collected and tested for IL-6, IL-8, and IL-10 as well as GM and BDG (in serum) levels in another single-center study of 106 prospectively enrolled adult patients with underlying hematological malignancy and suspected invasive pulmonary aspergillosis [65]. Elevated IL-6 and IL-8 levels from serum and BALF at the time of bronchoscopy were associated with increased 30-day all-cause mortality, and increasing IL-6 and IL-8 levels from serum within the first four days following bronchoscopy were associated with higher 90-day mortality rates [65]. In another study, stimulated peripheral blood cells from patients with IMI from Aspergillus spp. and Mucorales were associated with elevated genus-reactive CD4/CD69/CD154 positive T-cells, including elevated levels in 9 of 10 patients with proven IMI and levels were normal in 39/49 without proven IMI (sensitivity of 90% and specificity of 80%) [66]. Of note, the usefulness of this test was limited by low T-cell counts in patients with bone marrow suppression [66]. CD154 levels decreased in four patients with invasive *Mucorales* who underwent surgical resection, again suggesting that this marker may be useful in determining treatment response [67].

Further research is needed to evaluate the impact of different antifungals and different mechanisms of antifungal activity on the production of cytokines. In a recent in vitro model of invasive aspergillosis, cytokine levels increased by addition of hyphal suspension over 4 h about 54-fold for IL-6, and 1000-fold for IL-8. While conventional amphotericin B further increased IL-6 and, to a lesser extent, IL-8 levels, this was not the case for its liposomal formulation. Similarly, conventional amphotericin B substantially increased cytokines in blood without the presences of fungus, while fluconazole reduced cytokine increase for all three cytokines compared to stimulation with hyphae without antifungal agent [68]. Thus, antifungal agents may impact cytokine levels and this impact needs to be further evaluated and better understood prior to using cytokine levels as a criterion for monitoring treatment.

## 5. Conclusions

Immunologic markers, including cytokines and mold-reactive T cells, have shown promise for adding diagnostic value for diagnosis of IMI and may be attractive as markers for treatment monitoring and outcome prediction of IMIs, in particular those with negative serum GM levels. Multi-center studies are needed, however, to validate these findings, determine reliable cutoff levels for these assays, and assess whether immunologic markers can be readily and cost-effectively measured with easily usable assays with low turnaround time so that they are useful in guiding treatment. Future studies will also need to evaluate the potential of serial cytokine measurement as a predictor for outcomes and tool for treatment monitoring in IA and non-Aspergillus IMI, and determine if the change in serial cytokine levels is still predictable of IA and IMI outcome in individuals with complicating viral or bacterial infections. Finally, more research is needed to determine if immunologic markers can be incorporated into algorithms to identify patients who would benefit from stepdown in therapy (e.g., transition from intravenous to oral antifungals), cessation of therapy, or conversely the broadening of antifungal therapy.

## Figures and Tables

**Table 1:** Comparison of various reported biomarkers/cytokines in IMI diagnosis and treatment.

Sample	Biomarker/Cytokine	Mechanism of Action/Source	IMI Association?	Prognostic of Mortality?	Change with Treatment?
BALF	GM	Component of fungal cell wall	Elevated in IMI	Positive level at baseline and persistently positive/elevated level associated with increased mortality [30]	*Unknown (BALF often not resampled)*
	IL-6	Produced by alveolar macrophages and lung epithelial cells	Elevated in IMI [28,40]	Higher levels at time of sampling associated with increased mortality [65]	*Unknown*
	IL-8	Produced by alveolar macrophages and lung epithelial cells	Elevated in IMI [28,40], Sp/Sn >90%	Higher levels at time of sampling associated with increased mortality [65]	*Unknown*
	IL-17A	Secreted by Th17 cells [69], possibly also neutrophils [70]	Elevated in IMI [40]	*Unknown*	*Unknown*
	TNFα	Thought to be from Th17 cells, but other immune cells (e.g., macrophages, dendritic cells) may also contribute [25]	Elevated in IMI, but poor Sn/Sp [40]	*Unknown*	*Unknown*
	IL-1β	Secreted by activated macrophages [71]	Elevated in IMI [40]	*Unknown*	*Unknown*
	IL-23	Likely also from Th17 cells [69]	Elevated in IMI [40]	*Unknown*	*Unknown*
Serum	GM	Component of fungal cell wall	Elevated in IMI	Yes [65,72]	Decreases with successful treatment, increase should prompt clinical re-evaluation
	BDG	Component of fungal cell wall	Elevated in IMI	Yes [65,73]	Decreases with successful treatment, increase should prompt clinical re-evaluation
	IL-6	Produced by alveolar macrophages and lung epithelial cells	Elevated in IMI [28,40]	Higher levels at time of sampling associated with increased mortality [65]	Persistently elevated levels during serial weekly measurements associated with poor outcome [64]. Increasing levels after 4 days associated with higher 90-day mortality [65]
	IL-8	Produced by alveolar macrophages and lung epithelial cells	Elevated in IMI [28,40]	Higher levels at time of sampling associated with increased mortality [65]	Persistently elevated levels during serial weekly measurements associated with poor outcome [64]. Increasing levels after 4 days associated with higher 90-day mortality [65]
	IL-10	Produced by T cells, B cells, monocytes, and macrophages [74]	Elevated in IMI [75]	No [65]	Decreasing IL-10 within first 2 weeks of therapy associated with lack of response to IA treatment [64]. Not predictive of mortality [65]
	IL-17A	Secreted by Th17 cells [69], possibly also neutrophils [70]	Elevated in IMI [40]	*Unknown*	*Unknown*
	IL-23	Likely also from Th17 cells [69]	Elevated in IMI [40]	*Unknown*	*Unknown*
	CD4/CD69/CD154+ T cells	Bone marrow	Elevated in IMI [58]	*Unknown*	*Unknown*

Abbreviations: BALF, bronchoalveolar lavage fluid; BDG, (1,3)-β-D-glucan; GM, galactomannan; IL, interleukin; IMI, invasive mold infection; Sn, sensitivity; Sp, specificity; TNFα, tumor necrosis factor alpha.
